# Persistent organic pollutants and prostate cancer: multiple mechanisms and comprehensive control strategies

**DOI:** 10.3389/fcell.2026.1741752

**Published:** 2026-03-11

**Authors:** Chengsen Lv, Hongliang Cao, Shuxin Li, Hao Du, Lianchao Yang, Liming Wang, Shanyu Liu, Honglan Zhou, Jialin Gao

**Affiliations:** Department of Urology, The First Hospital of Jilin University, Changchun, China

**Keywords:** persistent organic pollutants, prostate cancer, endocrine disruptors, environmental pollutants, prevention, treatment

## Abstract

Persistent organic pollutants (POPs) are a class of chemical substances with environmental persistence, bioaccumulation, and high toxicity, which are widely present in the environment and food chains. Prostate cancer (PCa) is a highly prevalent malignant tumor in the male reproductive system, and the association between its incidence and POPs exposure has attracted increasing attention. This review systematically summarizes recent epidemiological and experimental research evidence, indicating that multiple POPs are associated with the incidence risk, invasive progression, and poor clinical outcomes of PCa. A comprehensive mechanism analysis framework is constructed to clarify that POPs are associated with the occurrence and development of Pca mainly through four synergistic biological pathways: as endocrine disruptors, they interfere with androgen and aryl hydrocarbon receptor signaling pathways; potentially inducing epigenetic reprogramming including DNA methylation, histone modification, and non-coding RNA expression; may contribute to abnormal reorganization of cellular energy metabolism, lipid, and amino acid metabolism; and being linked to oxidative stress, which may lead to damage to the antioxidant defense system and genomic instability. Based on the above understanding of the mechanisms, we further propose a comprehensive prevention and control strategy covering the entire chain, including multi-dimensional public health intervention measures from source emission reduction, transmission pathway interruption to protection of susceptible populations. Moreover, we integrate the POPs exposure assessment into the whole-process clinical management of PCa, including the practical pathways for prevention, diagnosis, treatment, and prognosis. This review not only deepens the understanding of the complex mechanisms through which POPs are associated with PCa, but also provides a crucial theoretical basis for formulating evidence-based, precise prevention and treatment strategies.

## 1 Introduction

Prostate cancer (PCa) is a hormone-dependent malignant tumor that occurs in the prostate gland epithelium and is the most common cancer of the male genitourinary system (Cao et al., 2024). Its incidence varies significantly between regions and ethnic groups, with developed countries generally having higher rates than developing countries (Sung et al., 2021). In recent years, the incidence of PCa has been on the rise, imposing a significant economic burden on families and society, severely impacting patients’ quality of life, and even leading to anxiety and depression (Belpomme and Irigaray, 2011). PCa often lacks typical symptoms in its early stages, but as it progresses, patients may experience symptoms such as difficulty urinating, hematuria, urinary incontinence, and bone pain. Most patients are diagnosed at an advanced stage (Obaed et al., 2022). PCa exhibits high biological heterogeneity (Bossio et al., 2024), with some cases being highly aggressive. Although the etiology of PCa remains incompletely understood, current evidence suggests that associated risk factors include hormonal imbalances, prolonged exposure to persistent organic pollutants (POPs), genetic factors, high-fat diets, and obesity (Ramis et al., 2011). Clinically, treatment options primarily include radical prostatectomy and androgen deprivation therapy (ADT). However, challenges such as difficult treatment in advanced stages, significant differences in survival rates, and progression to fatal metastatic castration-resistant PCa still remain. Therefore, early prevention of risk factors plays a crucial role in the prevention and treatment of PCa.

**FIGURE 1:**
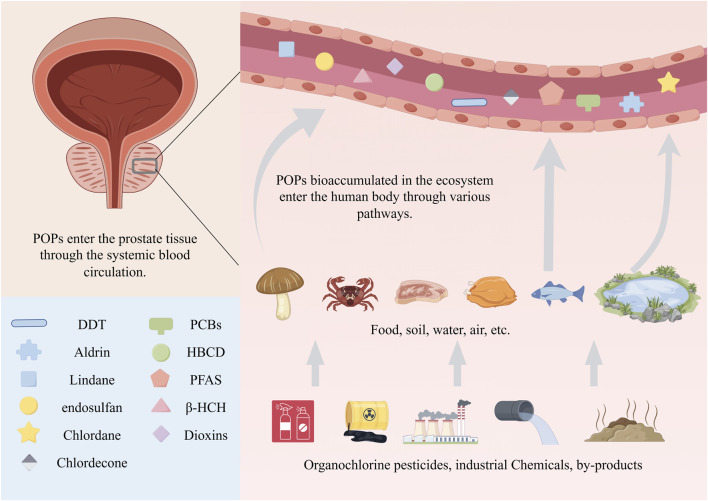
Multiple POPs are associated with PCa. The human body can be exposed to POPs in the ecosystem through various routes such as diet, respiration, skin contact, and mother-to-child transmission. After these pollutants enter the human body, they are distributed throughout the body via blood circulation. They are closely related to diseases of various systemic systems, and their association with PCa has attracted particular attention. POPs, persistent organic pollutants; PCa, prostate cancer; DDT, dichlorodiphenyltrichloroethane; β-HCH, β-hexachlorocyclohexane; PCBs, polychlorinated biphenyls; PFAS, per- and polyfluoroalkyl substances; HBCD, hexabromocyclododecane.

POPs refer to a class of organic chemical substances that are environmentally persistent, bioaccumulative, capable of long-range mobility, and highly toxic. They are environmental issues of global concern (Alavanja et al., 2003; El Balkhi and Saint-Marcoux, 2023). Environmental persistence, meaning they are difficult to break down naturally and can persist for long periods; bioaccumulation, which refers to their ability to accumulate in living organisms through the food chain (Ben et al., 2021); long-range mobility, meaning they can migrate through the atmosphere, water bodies, or migratory species and deposit in areas far from their sources; and high toxicity, posing significant risks to human health. Common POPs include organochlorine pesticides, industrial chemicals, and unintended by-products from industrial production or combustion processes (Dickhut et al., 2016; Karabiyik and Ilgaz, 2025). Humans can be exposed to POPs through various routes, including diet, respiration, skin contact, and mother-to-child transmission, with dietary intake being the primary source and mother-to-child transmission being an important route of exposure for infants (Pedroso et al., 2022). Once absorbed, POPs, due to their high lipophilicity, primarily accumulate in subcutaneous and visceral fat and are distributed throughout the body via the bloodstream (Charazac et al., 2022). The hazards of POPs exhibit significant delay and are closely associated with various systemic diseases. Research indicates that exposure to POPs, particularly during critical periods such as infancy or adolescence (Lopez-Espinosa et al., 2016; Tsai et al., 2015; Vested et al., 2013), can interact synergistically with environmental factors such as high-fat diets, contributing to reproductive health disorders and being associated with the development of various diseases including PCa, testicular cancer, liver cancer, polycystic ovary syndrome, endometriosis, and breast cancer (Boyd et al., 2022; Kahn et al., 2020). Among these, the relationship between POPs and PCa has drawn particular attention (Figure 1). Recent studies have found that multiple POPs can be detected and accumulate long-term in the adipose tissue of the human prostate (Antignac et al., 2023). They may participate in the pathological process of PCa by interfering with metabolic, endocrine, and epigenetic pathways, thereby affecting the normal physiological functions of the prostate. Notably, PCa and breast cancer, as two highly prevalent hormone-dependent cancers (Magnifico et al., 2025), share many etiological similarities, including hormone-regulated growth patterns, partially overlapping genetic susceptibility loci, and common vulnerabilities to environmental factors (Calvo Chozas et al., 2021; Coffey, 2001). Studies have shown that various POPs are closely associated with an increased risk of breast cancer (Fiolet et al., 2022). However, compared with breast cancer, the epidemiological evidence on the association between POPs and PCa has accumulated relatively late. In particular, the molecular mechanism network specific to prostate tissue has not been fully elucidated. This review aims to systematically summarize the latest evidence on the association between POPs and PCa, construct an integrated mechanism framework, and deeply explore the relationship between POPs and the occurrence and development of PCa.

## 2 Epidemiological and experimental evidence linking POPs to PCa

Recent observational studies have shown that multiple POPs play a role in the risk of developing PCa, its aggressive progression, and adverse clinical outcomes. The studies also observed a clear dose-response trend (Lemarchand et al., 2016; Lim et al., 2017). Meanwhile, several animal studies have further reported similar results, supporting the carcinogenic potential of POPs.

### 2.1 Organochlorine pesticides and PCa

Organochlorine pesticides are a class of organic compounds containing chlorine atoms. These pesticides were once widely used as pesticides in agriculture and public health due to their stable chemical properties, broad spectrum of insecticidal activity, long residual efficacy, and low production costs. Due to their long-term persistence in the environment and cumulative effects on organisms, many countries have strictly restricted or banned their use. Nevertheless, the harm of organochlorine pesticides to the human body persists (Andreotti et al., 2012; Koutros et al., 2013; Krstev and Knutsson, 2019). Consequently, farmers, as primary users and a highly exposed group, face an elevated risk of PCa (Lemarchand et al., 2016; Band et al., 2011; Lemarchand et al., 2017). Multiple studies have provided evidence for this (Table 1). In a case-control study comparing 173 individuals exposed to pesticides with 162 unexposed controls, it was found that environmental pesticide exposure both indoors and outdoors in agriculturally intensive regions was associated with PCa (Cockburn et al., 2011). A historical cohort study in Taiwan examining the relationship between dichlorodiphenyltrichloroethane (DDT) exposure and reproductive system cancer risk found, after 22 years of follow-up, that DDT was associated with PCa, and it was observed that each additional spraying of DDT significantly increased the cancer risk. This provides epidemiological evidence for the potential association between early DDT exposure and long-term carcinogenic risk (Chang et al., 2025), consistent with previous study results (Pi et al., 2016). Dichlorodiphenyldichloroethylene (DDE), the primary stable metabolite of DDT in the environment, is also associated with the development of PCa (Emeville et al., 2015). Another study on organochlorine pesticide residues in honey near abandoned pesticide storage facilities indicated that DDT, lindane, and endosulfan pose high potential risks to the health of both adults and children (Ben et al., 2021), and these substances have also been confirmed as risk factors for PCa in experimental animal models (El-Nahhal, 2020). A case-control study by Abhishek et al. (2020) also found that β-hexachlorocyclohexane (β-HCH) levels in the PCa group were associated with high-grade PCa. Men in the highest quartile of oxychlordane concentration in serum had twice the risk of metastatic PCa compared to those in the lowest quartile, suggesting that oxychlordane may play a role in metastatic PCa (Koutros et al., 2015). Additionally, biochemical recurrence after prostate resection has been a clinical challenge. A recent cohort study on chlordecone, which has estrogenic activity, found that the risk of biochemical recurrence significantly increased with rising plasma chlordecone concentrations, indicating that this pollutant negatively impacts PCa treatment outcomes (Brureau et al., 2020). These multiple studies from both population-based and animal experiments have consistently demonstrated the harmful effects of various types of organochlorine pesticides on PCa.

**TABLE 1:** Observational and experimental studies reveal the association between organochlorine pesticides and PCa.

Classification	POPs	Molecular formula	Authors, year	Study type	Sample size, dose	Results
Organochlorine pesticides	DDT	C_14_H_9_Cl_5_	Chang et al. (2025)	Left-truncated cohort study	n = 2,327,099	(1) Increased exposure is positively correlated with an increased risk of reproductive system cancers
	Chlordane	C_10_H_6_Cl_8_	Hurwitz et al. (2023)	Case-control study	n = 3,368	(1) May interact with specific genetic susceptibility variants for PCa. (2) Variants in genes involved in DNA damage repair and androgen regulation
	Endosulfan	C_9_H_6_Cl_6_O_3_S	Wa et al. (2020)	Experimental study	8.16 μg/mL	(1) Increased number of migrating and invading cells. (2) Effects on the expression of biomarkers and key proteins in signaling pathways
	Chlordecone	C_10_Cl_10_O	Legoff et al. (2021)	Experimental study	n = 83, 100 μg/kg body weight/day	(1) The prostate epithelial cells of mice proliferate in a clustered pattern. (2) The occupancy of histone marks changes. (3) The expression of genes related to hormone pathways, cell proliferation, self-renewal, and epigenetic regulation increases
	β-HCH	C_6_H_6_Cl_6_	Abhishek et al. (2020)	Case-control study	n = 279	(1) The content of β-HCH in the case group was 2 to 4-fold higher than that in the control group, and the ROS level was twice as high
	Aldrin	C_12_H_8_Cl_6_	Koutros et al. (2013)	Prospective cohort study	n = 54,412	(1) Increase the risk of aggressive PCa. (2) Have an interaction with a family history of PCa
	Lindane	C_6_H_6_Cl_6_	Band et al. (2011)	Case-control study	n = 5,152	(1) Significant excess risk observed at high exposure levels, with PCa risk nearly twofold higher

POPs, persistent organic pollutants; PCa, prostate cancer; DDT, dichlorodiphenyltrichloroethane; β-HCH, β-hexachlorocyclohexane; ROS, reactive oxygen species.

### 2.2 Industrial chemicals and PCa

Industrial chemicals are a broad category of chemical products distinct from agricultural chemicals, typically referring to various chemical substances used in industrial production, including basic chemical raw materials, flame retardants, fuels, dyes, cosmetics, detergents, and more. They play a crucial role in industrial production, are widely applied across multiple industries, and hold significant value. However, as a class of substances within POPs, industrial chemicals also pose irreversible health risks to humans. Both prospective cohort studies and case-control studies investigating the association between polychlorinated biphenyls (PCBs) and PCa have found that PCBs are associated with an increased risk of PCa (Table 2), suggesting they may play a role in the etiology of PCa (Lim et al., 2017; Pi et al., 2016; Ruder et al., 2014). To assess the association between dietary exposure to PCBs and the incidence and mortality rates of PCa at different grades, Ali et al. (2016) conducted a follow-up study on a cohort of 32,496 men, finding that PCBs exposure was positively correlated with the incidence of high-grade PCa and fatal PCa. Subsequently, at exposure levels relevant to the human body, they investigated the cell invasion and related markers induced by non-dioxin-like polychlorinated biphenyl congener 153 (PCB-153) in normal prostate stem cells and three different PCa cell lines (PC3, DU145, and 22RV1). They found that after exposure to PCB-153, the cell invasion ability and related markers, including MMP9, MMP2, Slug, and Snail, in human PCa cells and prostate stem cells increased significantly. In a dose-response meta-analysis involving 6,932 participants, it was found that for every 1 μg/g increase in PCBs concentration in individual lipids, the risk of PCa increased by 49%, indicating a positive correlation between PCBs concentration in the body and PCa risk (Lim et al., 2015). In addition, 2,2′,4,4′-tetra brominated diphenyl ether (BDE-47), one of the most predominant congeners of polybrominated diphenyl ethers (PBDEs), has also been reported to be associated with PCa in recent years. A study showed that low-dose BDE-47 can promote the proliferation, migration, and invasion of PCa cells *in vitro* and *in vivo* (Tian et al., 2024). Per- and polyfluoroalkyl substances (PFAS) are a broad class of widely used synthetic chemicals, including perfluorooctane sulphonate (PFOS) (Yang et al., 2025), perfluorohexane sulfonic acid (PFHxS) (Troeschel et al., 2024), and perfluorooctanoic acid (PFOA) (Hardell et al., 2014), which have been reported to be associated with the occurrence of PCa (Ohlander et al., 2022). A study on the association between PFAS and community cancer risk found that residents in communities exposed to PFAS had a higher risk of PCa than those in unexposed communities (Messmer et al., 2022). Based on the results of observational and experimental studies, exposure to industrial chemicals may play a role in the development of PCa. This provides valuable scientific evidence for the prevention of PCa.

**TABLE 2:** Observational and experimental studies reveal the association between industrial chemicals, by-products in production and PCa.

Classification	POPs	Molecular formula	Authors, year	Study type	Sample size, dose	Results
Industrial chemicals	PCBs	C_12_H_10-n_Cl_n_	Buñay et al. (2023)	Experimental study	10 ng/kg body weight/day	(1) Increase in the diameter and surface area of prostate organoids. (2) Cell migration capacity increased by 1.8-fold, and invasion capacity increased by 2.7-fold. (3) Increase in the number of lymph nodes. (4) Increased intra-cavitary spread of lesions in the posterior and anterior lobes of the prostate, with high-grade lesions widely distributed throughout each lobe
	PFAS	C_n_F_2n+1_-R	Hu et al. (2022)	Experimental study	10 mg/kg body weight/day	(1) The tumor growth in mice was greater at each time point, with an average volume of 710 mm^3^
	HBCD	C_12_H_18_Br_6_	Kim et al. (2016)	Experimental study	Dose based on biological relevance	(1) Enhance the cell migration ability. (2) Improve the cell transcription level
By-products in production	Dioxin	C_12_H_8-n_Cl_n_O_2_	Green-Lott et al. (2025)	Retrospective cohort study	n = 2,600,000	(1) Associated with PCa incidence, mortality, and adverse outcomes

POPs, persistent organic pollutants; PCa, prostate cancer; PCBs, polychlorinated biphenyls; PFAS, per- and polyfluoroalkyl substances; HBCD, hexabromocyclododecane.

### 2.3 By-products in production and PCa

In addition to organochlorine pesticides and directly produced industrial chemicals, certain by-products of production have also been identified as potential risk factors for PCa (Hoenemeyer, 2013; Mullins and Loeb, 2012). Agent Orange is a toxic chemical herbicide widely used during the Vietnam War, named for the orange stripes on its packaging drums. Its toxicity stems primarily from dioxins produced as by-products during its manufacturing process. Dioxins are not intentionally manufactured but primarily produced as by-products of industrial processes, waste incineration, and chemical production. Dioxins are a class of highly toxic tricyclic aromatic organic compounds with highly stable chemical properties, making them difficult to degrade in the environment. They can enter the human body through water sources and soil, accumulate in biological organisms over the long term, and have long-term impacts on human health and the ecological environment. In a large-scale cohort study on Agent Orange exposure among veterans, it was found that the exposed group had a higher incidence of PCa, mortality, and adverse outcomes compared to the unexposed group, suggesting that Agent Orange exposure may be associated with poorer PCa outcomes among veterans (Table 2) (Green-Lott et al., 2025). In another study aimed at determining the association between Agent Orange exposure among veterans and the risk of detecting high-risk PCa in the first prostate biopsy, the results showed that the increased risk of PCa associated with Agent Orange was primarily attributable to the increased risk of high-risk PCa in the first biopsy. These findings may contribute to improving PCa screening for veterans exposed to Agent Orange (Ansbaugh et al., 2013). In addition to Agent Orange, studies directly targeting dioxins have also reported their association with PCa. A study assessing the cancer burden attributable to dioxin exposure in the Chinese diet included participants from dietary surveys conducted in 31 provinces. They found that the disease burden of PCa was highest among men, and that dioxins and dioxin-like compound concentrations were highest in seafood across all food categories, suggesting that excessive dioxin exposure in coastal areas is a key concern. The study also compared trends in cancer burden risk over a decade, showing that the implementation of dioxin management policies reduced the cancer burden caused by dietary exposure to dioxins and dioxin-like compounds (Shi et al., 2024). In another study comparing dioxin exposure in hotspot and non-hotspot areas, it was found that in men in hotspot areas, levels of dihydrotestosterone (DHT), testosterone, and 3β-hydroxysteroid dehydrogenase (3β-HSD) activity significantly increased with age, suggesting that dioxin exposure may influence PCa by disrupting age-related hormone levels (Sun et al., 2017). In addition, two meta-analysis studies showed that exposure to dioxins and their homolog 2,3,7,8-tetrachlorodibenzo-p-dioxin (TCDD) was associated with an increased risk of PCa (K et al., 2018; Leng et al., 2014). These observational studies have noted a strong link between by-products generated and PCa. Compared with non-exposed populations, exposed populations have a higher risk of developing PCa.

### 2.4 Critical appraisal of the epidemiological evidence

The observational studies summarized above collectively underscore an association between exposure to POPs and PCa risk, particularly for aggressive and metastatic disease subtypes. However, a rigorous assessment of the methodological strengths and limitations inherent in this evidence base is crucial for evaluating the robustness of these associations and guiding future research. Cohort studies, which establish exposure status before disease onset, offer higher evidentiary value by minimizing recall bias and clarifying temporal relationships. In contrast, case-control studies Abhishek et al. (2020), while efficient for investigating rare outcomes, are more vulnerable to selection bias and differential recall of exposure history among cases versus controls. In addition to differences in study design, several methodological challenges also affect the consistency of the conclusions. Residual confounding of lifestyle factors, the interactions (such as synergistic or antagonistic effects) of mixed exposures to multiple POPs that are often not fully modeled. Inconsistent exposure assessment methods and definitions of PCa outcomes, and the heterogeneity of study populations in terms of genetic background and environmental co-exposures, may all lead to inconsistent effect estimates. It is worth noting that although most studies report a positive correlation between POPs and PCa risk, some studies in different populations show no or weak associations (Troeschel et al., 2024; Pourhassan et al., 2022). This reflects the multi-dimensional complexity of the exposure-disease relationship. In summary, current epidemiological evidence supports that multiple POPs are risk factors for PCa. However, establishing a clear causal inference still awaits future studies to further clarify the sources of heterogeneity and strengthen methodological rigor.

## 3 Multiple biological pathways of the impact of POPs on the incidence of PCa

**FIGURE 2:**
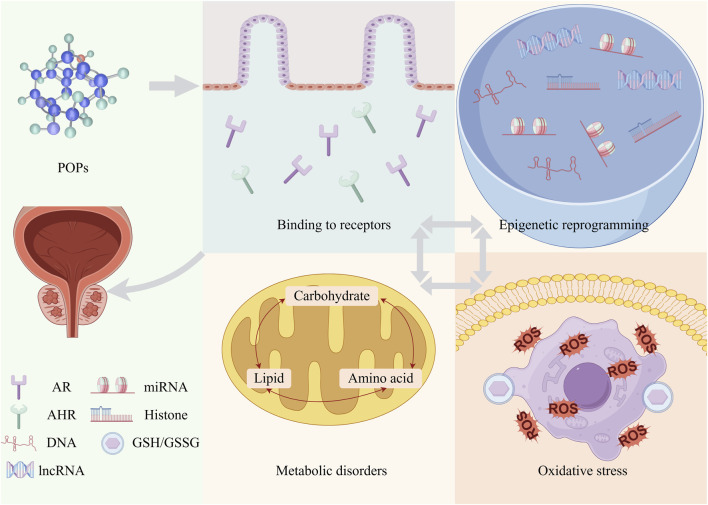
The potential mechanisms between POPs and PCa. These mechanisms include interacting with hormone receptors, inducing epigenetic reprogramming, triggering metabolic disorders, and activating oxidative stress pathways. They do not exist in isolation but are interrelated and act synergistically to promote the progression of PCa jointly. POPs, persistent organic pollutants; PCa, prostate cancer; ROS, reactive oxygen species; AR, androgen receptor; AHR, aryl hydrocarbon receptor; lncRNA, long non-coding RNA; miRNA, microRNA; GSH/GSSG, glutathione/oxidized glutathione.

Although current observational studies have indicated an association between POPs and PCa, the exact molecular mechanism is a multifaceted and intertwined synergistic process. This section aims to construct an integrated framework to analyze this process. First, POPs act as endocrine disruptors, interfering with key hormone receptors (such as AR and AHR) to initiate abnormal cellular signaling. These persistent abnormal signals may then lead to lasting epigenetic reprogramming, altering the gene expression of cells through DNA methylation, histone modifications, and non-coding RNA. Ultimately, these changes manifest as malignant cellular characteristics, supporting uncontrolled growth by reprogramming metabolic pathways (such as the Warburg effect and lipid/amino acid metabolic disorders) and triggering persistent oxidative stress, leading to DNA damage and genomic instability. These levels are not linear but form a synergistic network that jointly contributes to the initiation, progression, and metastasis of PCa (Figure 2) (Boyd et al., 2022).

### 3.1 Hormone interference

**FIGURE 3:**
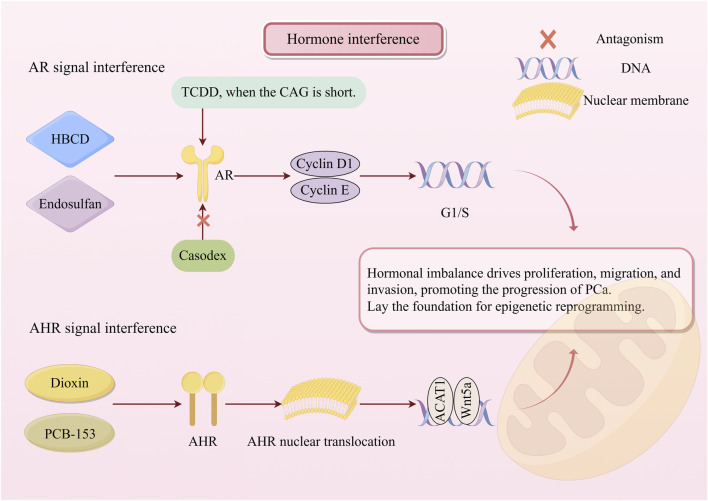
Two major pathways of hormone interference. POPs mimic hormones to activate the AR signaling or AHR signaling pathway, respectively, and accelerate the transition of the cell cycle from the G1 phase to the S phase. Then, by regulating the expression of specific target genes, they ultimately disrupt cell homeostasis and promote the progression of PCa. POPs, persistent organic pollutants; PCa, prostate cancer; HBCD, hexabromocyclododecane; TCDD, 2,3,7,8-tetrachlorodibenzo-p-dioxin; PCB-153, polychlorinated biphenyl congener 153; AR, androgen receptor; AHR, aryl hydrocarbon receptor.

Hormones are substances secreted by the endocrine system that can regulate a variety of physiological functions. POPs, as endocrine disruptors, can alter hormone levels in the body and have been associated with PCa (De Falco and Laforgia, 2021). Many pesticides can mimic or block the transcriptional activation mediated by endogenous hormones by binding to hormone receptors, thereby inducing an imbalance in cellular homeostasis (Figure 3) (Hurwitz et al., 2023). The androgen receptor (AR) signaling pathway is one of the core targets of POPs interference. The AR gene contains a variable CAG repeat sequence, whose length influences AR transcriptional activity. When the CAG repeat sequence is shorter, TCDD can abnormally activate AR in non-tumor prostate cells (PNT1A), altering hormone levels in the body (Björk and Giwercman, 2013). Hexabromocyclododecane (HBCD) activates the AR signaling pathway by mimicking the function of the natural androgen DHT, significantly enhancing the viability and migration capacity of PCa (LNCaP) cells; upregulates key cell cycle factors cyclin D1 and cyclin E, accelerates G1/S phase transition to drive cell proliferation; and inhibits pro-apoptotic genes, enhancing cell survival capacity. This carcinogenic effect can be reversed by Casodex, an AR antagonist, suggesting its AR pathway-dependent carcinogenic mechanism (Kim et al., 2016). Endosulfan, another androgen mimetic substance, can bind to AR and activate downstream signaling pathways, significantly enhancing AR protein expression and nuclear translocation in LNCaP cells at nanomolar concentrations (Singh et al., 2021).

The aryl hydrocarbon receptor (AHR) signaling pathway is another important pathway for POPs carcinogenesis. Studies have shown that exposure to POPs such as dioxins or PCB-153 activates the AHR, triggering its translocation from cytoplasmic diffuse distribution to nuclear aggregation. Activated AHR subsequently binds to transcription factor binding sites (TFBSs) in the highly conserved promoter sequence of the *ACAT1* gene. Activation of this gene has been observed in PCa and mouse models, leading to overexpression and accumulation of Acetyl-CoA Acetyltransferase-1 (ACAT1). This represents the key molecular mechanism driving the abnormal elevation of ACAT1 in PCa under POPs exposure (Buñay et al., 2023). Additionally, AhR exposed to dioxins can also induce the upregulation of Wnt5a, a critical regulatory factor in PCa progression. These AhR-mediated changes, encompassing ACAT1 overexpression, Wnt5a-induced transcriptional alterations, and enhanced cell migration and invasion capacity mediated by the AhR signaling pathway, may collectively promote disease progression in PCa cells (Hrubá et al., 2011). This sustained receptor activation not only directly regulates target genes but also sets the stage for stable epigenetic reprogramming.

### 3.2 Epigenetic reprogramming

**FIGURE 4:**
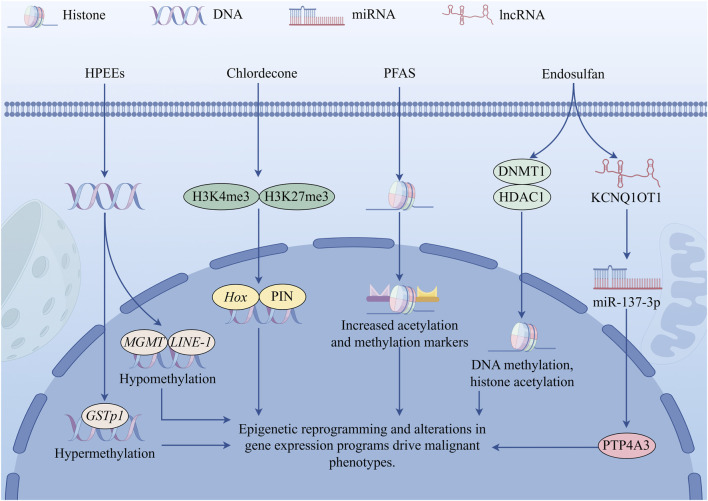
Three parallel mechanisms of epigenetic reprogramming. DNA methylation changes: Leading to abnormal hyper- or hypomethylation of the GSTp1 and MGMT gene promoters and LINE-1 repetitive elements. Histone modifications and transgenerational effects: Chlordecone can induce prostate abnormalities in F1 and F3, which is associated with an increase in H3K4me3 and a decrease in H3K27me3; Endosulfan induces the expression of DNMT1 and HDAC1 to participate in epigenetic regulation; PFAS causes an increase in multiple histone acetylation and methylation marks. Non-coding RNA regulatory axis: Endosulfan activates the KCNQ1OT1/miR-137-3p/PTP4A3 axis to induce EMT. These mechanisms together lead to changes in gene expression programs, disrupt cellular metabolism and redox homeostasis, and ultimately drive malignant phenotypes. HPEEs, high pesticide exposure events; PIN, prostatic intraepithelial neoplasia phenotype; F1, offspring; F3, third generation; DNMT1, DNA methyltransferase 1; HDAC1, histone deacetylase 1; PFAS, Per- and polyfluoroalkyl substances; EMT, epithelial-mesenchymal transition; lncRNA, long non-coding RNA; miRNA, microRNA.

Non-mutagenic epigenetic pathways play a significant role in the biological effects of POPs, and research in PCa has made notable progress. This epigenetic reprogramming primarily occurs through DNA methylation, histone modifications, and non-coding RNA regulation (Figure 4). DNA methylation alterations: Studies on male pesticide applicators have found that experiencing high pesticide exposure events (HPEEs) is associated with elevated DNA methylation levels at specific CpG sites in the *GSTp1* gene promoter region. Stratified analysis revealed that this *GSTp1* hypermethylation was more pronounced in older applicators and those with lower plasma folate levels. Additionally, in older applicators with lower folate levels, HPEEs also led to hypomethylation of the *MGMT* gene promoter region and the *LINE-1* repetitive element (Rusiecki et al., 2017).

Histone modification and transgenerational effects: In a mouse model study revealing the transgenerational epigenetic effects of POPs, maternal exposure to chlordecone during pregnancy not only directly affected the prostate health of their offspring (F1) male mice, but also led to prostate abnormalities in the third generation (F3) male mice that were not directly exposed, suggesting transgenerational effects. Specifically, F1 and F3 male mice exhibited increased expression of prostatic intraepithelial neoplasia phenotype (PIN), *Hox* genes, and self-renewal-related genes. This transgenerational effect is closely associated with epigenetic changes in prostate tissue, particularly increased histone H3K4me3 and decreased H3K27me3 (Legoff et al., 2021). In addition to activating the classical androgen signaling pathway as a mimetic ligand of the AR to promote the proliferation of LNCaP cells, endosulfan binds to the key epigenetic regulatory enzymes DNA methyltransferase 1 (DNMT1) and histone deacetylase 1 (HDAC1), and enhances their protein expression levels in LNCaP cells. This induction of DNMT1 and HDAC1 suggests that endosulfan can influence gene expression by regulating DNA methylation and histone modifications (Singh et al., 2021). Increased levels of multiple histone acetylation and methylation markers were detected in cells exposed to PFAS, consistent with previous reports from various laboratories describing PFAS exposure-associated changes in genome-wide or specific histone site methylation (Imir et al., 2021; Rashid et al., 2020; Tian et al., 2012; Wan et al., 2010; Wen et al., 2020).

Non-coding RNA regulation: Endosulfan mainly activates TGF-β signaling through the KCNQ1OT1/miR-137-3p/PTP4A3 axis to drive migration and invasion of PCa cells. Endosulfan activates the long non-coding RNA (lncRNA)-KCNQ1OT1, primarily located in the cytoplasm. KCNQ1OT1 competitively binds to the microRNA (miRNA)-miR-137-3p, relieving the inhibitory effect of miR-137-3p on the expression of protein tyrosine phosphatase 4A3 (PTP4A3), leading to increased PTP4A3 expression. Elevated PTP4A3 activates the TGF-β signaling pathway, thereby inducing epithelial-mesenchymal transition (EMT), manifested by downregulation of E-cadherin and upregulation of markers such as fibronectin and vimentin. Functional experiments confirmed that inhibiting KCNQ1OT1, overexpressing miR-137-3p, or inhibiting the PTP4A3/TGF-β signaling pathway can effectively reverse the EMT changes and enhance cell migration and invasion capacity induced by endosulfan (Wa et al., 2020; Wang et al., 2022).

In conclusion, multiple POPs induce abnormal DNA methylation, alter histone modification states, and regulate non-coding RNA networks, ultimately driving epigenetic reprogramming that promotes tumor growth, constituting a key non-genotoxic mechanism underlying PCa development under POPs exposure. These functional shifts are prominently manifested in cellular metabolic reprogramming, which we discuss next.

### 3.3 Metabolic disorders

Exposure to POPs has been associated with various cellular and systemic metabolic disorders, and metabolic changes are one of the key mechanisms linking POPs to PCa. Multiple POPs may synergistically contribute to metabolic reprogramming in PCa cells by restructuring energy metabolism, abnormally activating lipid metabolism, and reprogramming amino acid metabolism, thereby promoting malignant transformation and tumor progression.

Energy metabolism restructuring and pre-metastatic programs: Dioxins and PCB-153 mediate the reprogramming of the ketone body metabolic pathway by abnormally activating *ACAT1*. They not only upregulate the expression of *ACAT1* but also simultaneously activate key genes such as *OXCT1*, *BDH1*, and *HMGCL*, constructing a metastasis-promoting metabolic network in advanced PCa and directly driving the reconstruction of the tumor energy utilization pattern and the development of the pre-metastatic program. Inhibiting *ACAT1* can block the migration of cancer cells induced by pollutants, confirming the pro-cancer effect of this pathway (Buñay et al., 2023). In addition, Signal transducer and activator of transcription 3 (STAT3) serves as both a convergence point for diverse signaling pathways and a key regulator of cellular energy metabolism. β-HCH drives STAT3 activation, which in turn directly reprograms the glycolytic pathway by upregulating the expression of hypoxia-inducible factor-1α (HIF-1α) and pyruvate kinase isozyme M2 (PKM2) (Rubini et al., 2018). This metabolic reprogramming induces the typical Warburg effect, which manifests as enhanced lactate production as an energy source even under normoxic conditions, rather than the more efficient mitochondrial oxidative phosphorylation pathway, changing the energy metabolism pattern of tumor cells and leading to energy metabolism imbalance in tumor cells. This effect serves as an adaptive mechanism to support the synthetic demands of uncontrolled proliferation in cancer cells (Epstein et al., 2017). In PCa cells, PFAS exposure, especially in combination with a high-fat diet, induces metabolic phenotype alterations, enhances glucose metabolism through the Warburg effect, increases pyruvate production, and upregulates the pyruvate dehydrogenase complex (PDC), leading to elevated acetyl-CoA levels (Imir et al., 2021). Concurrently, PFAS can also promote the biosynthesis of phosphatidylethanolamine, the degradation of homocysteine, the degradation of lysine, and biotin metabolism, further reinforcing its impact on acetyl-CoA metabolism (Chen et al., 2021; Leav et al., 2010). These metabolic changes, which enhance glycolysis, oxidative phosphorylation, the citric acid cycle, and the pentose phosphate pathway, push cells toward a more energy-efficient and mitochondria-dependent state. They work together with the peroxisome proliferator-activated receptor alpha (PPARα) signaling pathway, which is activated by PFAS, to contribute to the proliferation of PCa cells and tumor growth (DeWitt et al., 2009). Similar metabolic reprogramming is also observed in exposure to other POPs. For example, the brominated flame retardant BDE-47 can enhance glycolysis and lactic acid production in PCa cells by upregulating Topoisomerase II Alpha (TOP2A) and Lactate Dehydrogenase A (LDHA) (Tian et al., 2024). This study directly links lactate metabolism to epigenetic modification and finds that accumulated lactate can catalyze the lactylation of histone H3K18, thereby forming an epigenetic-metabolic positive feedback loop that promotes the expression of oncogenes.

Lipid metabolism abnormalities: PFAS exposure has been shown to disrupt multiple lipid metabolism pathways at both the cellular and systemic levels, including changes in fatty acid, linoleic acid metabolism, and glycerophospholipid metabolites (Alderete et al., 2019). Exposure to aldrin can induce increases in certain types of triglycerides. This suggests that specific lipid compounds and their associated metabolic pathways may be deeply involved in the process by which PCa cells acquire a malignant phenotype following POPs exposure (Bedia et al., 2015).

Amino acid metabolic reprogramming: PFAS alter energy metabolism in mouse livers, affecting amino acid metabolism, such as aspartic acid, asparagine, tyrosine, arginine, proline, and the citric acid cycle, while upregulating peroxisomal β-oxidation regulatory enzymes and increasing the concentration of related organelles (Costello and Franklin, 2006; Domazet et al., 2016; Tan et al., 2012). In prostate stem-progenitor cells (SPCs), PFAS activate peroxisome proliferator-activated receptors (PPARs) and retinoid X receptors (RXRs), driving cellular glycolytic pathways and serine and glycine metabolism, directly promoting enhanced stem cell self-renewal capacity and abnormal differentiation (Hu et al., 2022). Metabolomics analysis has provided evidence that POPs-mediated extensive metabolic reprogramming collectively constitutes an important metabolic mechanism through which POPs exposure increases the risk of PCa. Notably, the active metabolic intermediates (such as acetyl-CoA) produced during metabolic reprogramming can themselves serve as substrates or cofactors for epigenetic modification enzymes, thereby affecting the epigenetic state (Chisolm and Weinmann, 2018), forming a feedback loop. Meanwhile, metabolic abnormalities, especially mitochondrial dysfunction, are the main source of intracellular reactive oxygen species (ROS), which are directly linked to the oxidative stress pathway (Riou and Geny, 2025).

### 3.4 Oxidative stress

Oxidative stress is not an isolated event. As mentioned above, it can be directly triggered by metabolic disorders. Meanwhile, the AR/AHR signaling directly activated by POPs can also upregulate pro-oxidant enzyme systems. Conversely, DNA damage and protein modifications induced by ROS can affect transcription factor activity and epigenetic regulation, forming a cycle that accelerates malignant transformation. Abnormal activation of the AR signaling pathway not only promotes excessive production of ROS but also contributes to oxidative stress by reducing the activity of free radical scavenging enzymes and lowering intracellular antioxidant levels (Gonthier et al., 2019), leading to an imbalance in redox homeostasis. This state is characterized by the impairment of the ability to maintain redox homeostasis, which in turn damages the functions of biomolecules (Sies et al., 2017). The core regulatory mechanism of oxidative stress relies on the redox cycle of glutathione in the cytoplasm and mitochondria. Glutathione is crucial for protecting mitochondria from ROS generated by the electron transport chain (Kowluru and Mishra, 2015). However, in PCa patients, the expression of glutathione peroxidase (GPx) is significantly reduced, weakening glutathione’s ability to neutralize mitochondrial ROS. Further depletion of the glutathione system exacerbates the pro-oxidative environment, driving abnormal gene expression and malignant transformation. Meanwhile, excessive ROS induces mutations and genomic instability by oxidizing cell membranes, modifying proteins, and directly damaging DNA structures, ultimately promoting tumorigenesis. Notably, POPs can synergistically amplify DNA damage risks by inducing ROS bursts and oxidative stress in conjunction with androgen stimulation. Specifically, β-HCH may increase cellular ROS levels, leading to the accumulation of oxidized glutathione (GSSG), a decrease in the reduced glutathione/oxidized glutathione (GSH/GSSG) ratio, and alterations in the redox state of glutathione, which is a reliable marker of oxidative stress (Rubini et al., 2020). In addition, *in vitro* research evidence indicates that PFAS can induce dose-dependent DNA strand breaks under high-concentration exposure conditions (Wielsøe et al., 2015). Genetic studies also support the role of oxidative stress in PCa, such as the association between variants at the *CAT* rs1001179 locus of oxidative stress-related genes and disease risk (Martinez-Gonzalez et al., 2020). In summary, Oxidative stress induced by POPs exposure serves as a key link connecting abnormal androgen signaling, defects in the endogenous antioxidant system, and impaired DNA damage repair, playing a crucial role in the initiation and progression of PCa (Koutros et al., 2011).

### 3.5 Gaps in mechanism cognition and future directions

Despite significant progress in elucidating the potential pathways through which POPs are associated with PCa, key knowledge gaps still remain. First, most of the evidence comes from studies on single POPs; however, human exposure involves long-term, complex mixtures of POPs. Little is known about the interactions of these mixtures. Second, the cross-talk and hierarchical relationships between different major mechanistic axes have not been fully elucidated. For example, how POPs-induced changes in hormone levels feedback to affect the epigenetic state needs to be further dissected using integrated multi-omics approaches. Third, although animal models provide strong mechanistic plausibility, data on the impact of prenatal or early-life exposure to POPs on the development of PCa in adulthood in humans are still scarce. Filling these gaps is crucial for a comprehensive understanding of POPs as environmental carcinogens.

## 4 Comprehensive public health intervention measures for POPs

**FIGURE 5:**
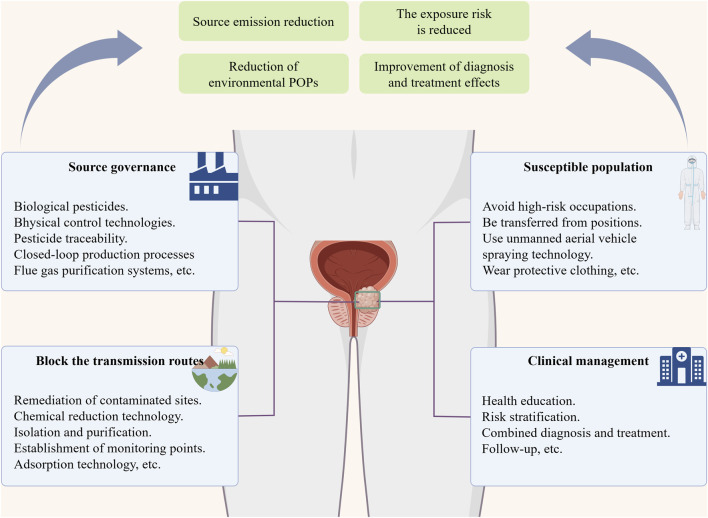
Multi-dimensionally address the threats of POPs to PCa. Establishing a comprehensive public health intervention system and a full-course clinical management mechanism for POPs simultaneously can effectively reduce the exposure risk of the population and improve the diagnosis and treatment effects. POPs, persistent organic pollutants; PCa, prostate cancer.

To effectively prevent and control the severe challenges posed by POPs to PCa, single-pronged governance measures are no longer sufficient. There is an urgent need to establish a comprehensive public health intervention system that covers the entire chain. The core of this system lies in implementing multi-dimensional, synergistic strategies. Eliminating the production and release of POPs at the source is the fundamental defense; interrupting their migration, dispersion, and bioaccumulation in environmental media along transmission pathways is a critical step in effectively blocking the entry of pollutants into the human body; and implementing targeted protection for populations with specific genetic susceptibilities or high exposure risks is the final line of defense in reducing individual disease risks (Figure 5). This comprehensive governance model, which transcends end-of-pipe treatment or individual protection thinking and combines source control, transmission interruption, and protection of susceptible populations, is the only way to systematically reduce POPs exposure loads in populations, lowering the incidence risk and disease burden of POPs-related PCa.

### 4.1 Source governance

Managing POPs at their source to reduce their harmful effects on PCa is of fundamental and irreplaceable significance. The inherent characteristics of POPs determine this. These characteristics make the subsequent governance face extremely high costs, heavy economic burdens, and limited effectiveness (Nedellec et al., 2016). Source management is the only effective strategy to prevent POPs from entering the environmental cycle, curb their bioaccumulation and amplification effects, address global pollution challenges, and prevent widespread toxic hazards. The historical lessons are profound. The Guadeloupe region suffered from persistent soil contamination due to the use of chlordecone in banana plantations, leading to long-term exposure through the food chain (Pochet et al., 2025); proactive practices have also proven their effectiveness, as dioxin management policies have significantly reduced the cancer burden related to exposure to dioxins and dioxin-like compounds (Shi et al., 2024). Source management requires targeted strategies for different sources of POPs. For pesticide-based POPs, their production and use in agriculture and public health should be banned entirely. Safer and more environmentally friendly alternatives, such as the promotion of biological pesticides and physical control technologies, should be adopted to reduce environmental residues and establish a traceability system for pesticide use. For industrial chemicals, production and circulation of materials containing POPs should be strictly restricted, perfluorinated compounds should be phased out, clean production processes should be promoted, and safe disposal standards for POPs in waste such as electrical equipment and fluoropolymer products should be established. Closed-loop production processes should be adopted to prevent POPs from entering the environmental cycle. For unintentionally produced by-products, optimize industrial combustion and chemical production processes, adopt advanced pollution control technologies such as efficient flue gas purification systems, and strengthen emission regulation in industries such as waste incineration and metal smelting to suppress their release at the source.

### 4.2 Block the transmission routes

To block the harm of POPs on PCa through transmission pathways, systematic interventions are needed in soil, water, and air. For soil media, remediation should be carried out in key contaminated sites, such as areas where pesticides have been used in the past, and *in situ* chemical reduction technology should be used to degrade residues and reduce their carcinogenic potential. Studies have shown that the proangiogenic activity of the dechlorinated derivatives of chlordecone is lower than that of chlordecone itself (Legeay et al., 2018). Contaminated farmland should be isolated and purified, and non-edible crops should be planted, or hydroponic technology should be used to block POPs from entering the food chain through edible crops. At the same time, hyperaccumulating plants can be introduced to accelerate the biodegradation of pollutants such as PCBs. For water pollution control, the key is to block exposure routes in drinking water and aquatic products. To ensure drinking water safety, water treatment plants should adopt advanced treatment processes such as activated carbon adsorption combined with reverse osmosis to reduce POPs concentrations in drinking water below the detection limit, thereby lowering the risk of PCa. Koutros et al. (2015) found that the concentration of oxychlordane is related to metastatic PCa. Dioxin concentrations in aquatic products are relatively high, making them a primary dietary exposure source (Watzl et al., 2012). Aquatic exposure management should be strengthened by establishing monitoring points in estuaries and coastal areas with severe POPs deposition and restricting the circulation of highly contaminated fish and shellfish. Artificial wetland buffer zones can also be constructed around farmland to utilize the adsorption capacity of aquatic plants to retain pesticide pollutants in runoff. In terms of airborne control, vegetation cover should be implemented at contaminated sites to reduce the wind-driven migration of volatile pesticides. Adsorption technology can be employed to reduce the concentration of POPs in the air and minimize human exposure risks. Studies indicate that the risk of PCa among residents within a 12-km radius of industrial zones is correlated with the spatial distribution of POPs exposure (Ramis et al., 2011). These intervention measures aim to reduce the environmental presence and human exposure to POPs by addressing transmission pathways.

### 4.3 Susceptible population

Susceptible populations are more sensitive to the health effects of POPs due to their specific genetic and metabolic foundations. By identifying environmental and occupational exposure risks and strengthening targeted protective measures, the risk of diseases such as PCa can be reduced. Precision protection strategies must be closely aligned with individual risk characteristics. For susceptible individuals engaged in agriculture, exposure to pesticides such as DDT and lindane has been associated with genetic damage. Abhishek and Barry et al. found that individuals carrying susceptibility genes such as *CYP1A1* rs1048943 or *ERCC1* rs2298881 have an increased risk of PCa when exposed to pesticides (Abhishek et al., 2020; Barry et al., 2012). To reduce risk, genetic screening can be used to identify high-risk individuals and prevent them from engaging in high-risk occupations involving pesticide spraying or those related to POPs. When necessary, individuals carrying high-risk genotypes should be transferred away from positions involving direct exposure. The use of drone spraying technology should be widely promoted, and protective clothing with activated carbon adsorption functionality should be mandated. A detailed occupational exposure record system should be established.

As the core of protection, pregnant women and infants must strictly avoid food and water sources in contaminated areas and prioritize the use of purified drinking water. Prenatal exposure to POPs can cause irreversible damage during critical developmental windows (Gore et al., 2015; Sanchez de Badajoz et al., 2017), permanently altering the epigenetic programming of the prostate. Exposure of pregnant women to POPs such as chlordecone increases the risk of gestational hypertension, premature birth, and low birth weight, which may be associated with delayed cognitive and motor development in offspring and increasing the risk of PCa in future generations, especially when there is a family history (Maudouit and Rochoy, 2019). Individuals with a high family history of PCa are considered high-risk, and their serum POP levels are positively correlated with disease risk and severity. For example, each 1 μg/g lipid increase in total PCBs is associated with a 49% increase in PCa risk (Lim et al., 2015). Therefore, their serum POP levels should be regularly monitored (Ali et al., 2016). The establishment and implementation of such a comprehensive system integrating genetic screening, exposure monitoring, and limit control, engineering protection, and personal protection is essential to block the carcinogenic cascade reaction of POPs. Its necessity is rooted in the irreversible damage of POPs to critical developmental windows, the clear dose-response relationship of occupational exposure, and the heavy health and economic burden of related diseases (Legoff et al., 2021; Xu et al., 2016).

## 5 Clinical whole-course management of POPs and PCa

POPs are modifiable environmental risk factors for PCa. Clinical practice requires a multifaceted approach, integrating POPs exposure into the entire management cycle of PCa and restructuring the diagnostic and treatment pathway (Figure 5). In prevention strategies, dietary intervention measures aimed at reducing exposure to POPs should be implemented (Quagliariello et al., 2017). Additionally, exposure restriction recommendations based on genetic background should be provided for pesticide-exposed individuals carrying DNA repair gene mutations (Cypriano et al., 2017). Concurrently, health education efforts should be intensified for the general public, particularly high-risk populations with a family history of PCa, to inform them about the sources of POPs and their potential carcinogenic risks. Through guidance on adjusting lifestyles and adopting key protective measures, their self-protection capabilities should be enhanced. Recent studies indicate that reducing the ratio of omega-6 to omega-3 fatty acids intake (N-6/N-3) in the diet may have a protective effect on pesticide-exposed populations (Sadeghi et al., 2023); supplementation with Tomato and Olive Bioactive Compounds (TOBC) can antagonize the activation of carcinogenic signaling pathways induced by β-HCH (Rubini et al., 2021).

Assessing environmental exposure risks, it is necessary to systematically collect patients’ environmental and occupational exposure histories, with a focus on exposure to pesticide-related and industrial-related POPs (Cypriano et al., 2017). Agricultural workers, residents of polluted areas, and populations in industrial zones should be classified as high-risk groups, and long-term exposure histories should be incorporated into PCa screening stratification management strategies to initiate or strengthen prostate-specific antigen (PSA) monitoring in advance (Hurwitz et al., 2025; Tan and Norhaizan, 2021). Meanwhile, the development of cell imaging technology based on supramolecular fluorescent probes can be explored for the screening of POPs exposure (Tang et al., 2018). Enhancing diagnosis and treatment decisions, caution should be exercised regarding the potential for DDT metabolites to inhibit PSA expression, leading to false-negative results and misleading clinical diagnoses (Wong et al., 2015). For exposed individuals with suspected symptoms but normal PSA levels, further evaluation should be conducted using digital rectal examination or imaging (Sun et al., 2017). Additionally, the potential of hydroxylated PCBs, especially 3′-OHCB28, as an adjunct therapy should be explored. By synergistically inhibiting the expression of heat shock protein 27 (HSP27), they can enhance the antiproliferative effect of abiraterone on PCa PC3 cells, reduce the required effective concentration, and provide a new treatment approach for drug-resistant patients (Daragan et al., 2020).

In terms of prognostic survival management, attention should be paid to the significantly increased risk of biochemical recurrence in exposed patients after surgery (Brureau et al., 2020), and a more intensive follow-up program should be developed. At the same time, the association between exposure to multiple POPs and high-grade, metastatic PCa should be recognized, and animal experiments have suggested that it promotes metastasis through *ACAT1* gene upregulation. Such patients may benefit from *ACAT1* gene testing to assess invasion risk (Koutros et al., 2015; Ali et al., 2016; Buñay et al., 2023).

Through the interdisciplinary collaboration network of clinical and environmental health, incorporate health monitoring in polluted areas into the public health system, promote the implementation of occupational protection education policies for high-risk groups, and build a comprehensive prevention and control chain from exposure assessment to precise intervention.

## 6 Conclusion and outlook

Accumulating evidence underscores a significant association between exposure to various POPs and the risk, aggressiveness, and poor prognosis of PCa. This review consolidates observational and experimental findings to propose an integrated mechanistic framework. These pollutants are associated with prostate carcinogenesis through several interconnected pathways: by disrupting endocrine signaling via androgen and aryl hydrocarbon receptors, by contributing to epigenetic reprogramming through alterations in DNA methylation, histone modifications, and non-coding RNA networks, by inducing metabolic dysregulation of energy, lipid, and amino acid metabolism, and by fostering oxidative stress and genomic instability. Based on this mechanistic understanding, comprehensive strategies are imperative. Effective mitigation requires comprehensive public health interventions targeting pollution sources, environmental transmission routes, and protection of high-risk populations. Simultaneously, clinical management of PCa must evolve to incorporate assessment of exposure history into prevention, diagnostic, therapeutic, and prognostic decisions. However, current research still has limitations. In the future, it is necessary to clarify the comprehensive effects of various POPs, decipher cross-talk between mechanistic axes, and validate findings from animal models in large-scale human cohorts. Biomarkers for exposure assessment and risk stratification should be developed, and therapeutic interventions targeting the activation pathways of POPs should be explored. Ultimately, addressing the persistent challenge of POPs-related PCa requires sustained interdisciplinary collaboration to translate scientific evidence into effective public health policies and clinical practices.
